# Strengthening nursing role and functions in stroke rehabilitation 24/7: A mixed‐methods study assessing the feasibility and acceptability of an educational intervention programme

**DOI:** 10.1002/nop2.202

**Published:** 2018-09-19

**Authors:** Mia Ingerslev Loft, Ingrid Poulsen, Bente Martinsen, Lone Lunbak Mathiesen, Helle Klingenberg Iversen, Bente Appel Esbensen

**Affiliations:** ^1^ Department of Neurology Rigshospitalet Glostrup Denmark; ^2^ Research Unit on Brain Injury Rehabilitation Copenhagen (RuBRIC), Clinic of Neurorehabilitation TBI Unit Rigshospitalet Hvidovre Denmark; ^3^ Department of Nursing Science, Institute of Public Health Aarhus University Copenhagen Denmark; ^4^ Clinical Research, Faculty of Health and Medical Sciences University of Copenhagen Glostrup Denmark; ^5^ Copenhagen Centre for Arthritis Research (COPECARE) Centre for Rheumatology and Spine Diseases VRR, Head and Orthopaedics Centre, Rigshospitalet Glostrup Denmark; ^6^ Faculty of Health and Medical Sciences, Department of Clinical Medicine University of Copenhagen Glostrup Denmark

**Keywords:** acceptability, behaviour change, complex intervention, educational intervention, feasibility, nursing, nursing role, rehabilitation, stroke

## Abstract

**Aim:**

To assess the feasibility of a nursing educational intervention for inpatient stroke rehabilitation and its acceptability from the nursing staff’s perspective.

**Background:**

There is currently a lack of interventions that integrate the diversity of nurses’ role and functions in stroke rehabilitation and explore their effect on patient outcomes.

**Design:**

We used a convergent, parallel, mixed‐method design with data interviews and questionnaires.

**Methods:**

Data collection was undertaken between February ‐ July 2016. Data from questionnaires (*N* = 31) were analysed using descriptive statistics. The interviews (*N* = 10) were analysed using deductive content analysis.

**Results:**

There was a high level of satisfaction with the educational programme in terms of its acceptability and feasibility. The qualitative findings disclosed the nursing staff's experiences with the educational programme. Mixed‐methods analysis showed confirmatory results that were convergent and expanded. Only minor adjustments are required before an effect study can be conducted.

## 1 INTRODUCTION

This paper describes a feasibility test of a stroke nursing educational intervention, *Rehabilitation 24/7*. The objective was to strengthen the roles and functions of nursing staff working in inpatient stroke rehabilitation.

Nurses have been described as key players in interdisciplinary inpatient stroke rehabilitation teams (Booth, Hillier, Waters, & Davidson, 2005; Langhorne, Williams, Gilchrist, & Howie, 1993). Being present 24/7, they have unique opportunities to influence inpatient stroke rehabilitation (Kirkevold, 2010). However, nurses have struggled to clarify their role and functions in inpatient stroke rehabilitation (Aadal, Angel, Dreyer, Langhorn, & Pedersen, 2013; Clarke, 2013; Kirkevold, 2010; Long, Kneafsey, Ryan, & Berry, 2002). One way of strengthening the nurses’ role and functions may be to develop interventions that integrate the diversity of their role and functions so that the effects on patient outcomes can be explored. However, studies that seek to maximize the contribution of nursing staff to inpatient stroke rehabilitation are almost non‐existent (Clarke, 2013).

## 2 Background

Stroke has major consequences for patients and relatives. Moreover, it affects people in different ways depending on prior functional and cognitive levels and the severity and duration of poststroke impairment. Stroke also influences physical, emotional, cognitive and social well‐being (Jennum, Iversen, Ibsen, & Kjellberg, 2015; Kvigne & Kirkevold, 2003).

Patients admitted to inpatient stroke rehabilitation describe the nursing staff in positive terms, but also as having imperceptible roles and functions in terms of their therapeutic contribution to the rehabilitation process (Hole, Stubbs, Roskell, & Soundy, 2014; MacDuff, 1998; Secrest & Thomas, 1999). Patients perceive nursing staff as only focusing on meeting patients’ basic physical needs, which gives patients a feeling of physiological and emotional isolation during their inpatient rehabilitation (Gallacher et al., 2013; Hole et al., 2014; Satink et al., 2013; Secrest & Thomas, 1999). Patients admitted to inpatient rehabilitation are described as being inactive and alone during the day (Bernhardt, Dewey, Thrift, & Donnan, 2004; West & Bernhardt, 2012) despite evidence that early rehabilitation and intensive training are significant for functional outcome (Askim, Bernhardt, Salvesen, & Indredavik, 2014; Bernhardt, Godecke, Johnson, & Langhorne, 2017). Research shows that patients’ interaction with healthcare professionals and their active involvement in their own rehabilitation are important for regaining skills and self‐esteem (National Health Board, 2011). Based on empirical studies, Kirkevold developed a theory about the nurse’s role in neuro‐rehabilitation of people who suffered a stroke. The study was developed in 1997 and revised in 2010 by integrating newer research of the nursing role and function and experience‐based knowledge from studies on patients’ recovery and adjustment process (Kirkevold, 1997, 2010 ). Her theory identified four therapeutic functions: “the conservative, the interpretative, the consoling and the integrative role and function in addition to a coordinating and leading function. Nurses facilitate bodily rehabilitation through conserving bodily functions, supporting the patients in continuing multiple therapies and helping patients interpret and integrate new learning skills into their everyday activities” (Kirkevold, 2010).

Despite the demand for interventions aimed at strengthening nurses’ role and functions in inpatient stroke rehabilitation, we only identified four such studies (Booth et al., 2005; Burton & Gibbon, 2005; Forster et al., 1999; Jones et al., 1998).In 1998, Jones et al. reported a quasi‐experimental study that involved a two‐hour classroom course aimed at improving nurses’ knowledge of and practice in the area of positioning patients. The authors concluded that the intervention had some effect (Jones et al., 1998). In 1999, Foster et al. reported on the effect of a physiotherapist‐led training programme concerning the attitudes of nurses caring for patients after stroke. The intervention consisted of nine hours of training, including lectures and interactive practical sessions. The effects were measured using an attitude questionnaire and qualitative interviews (Forster et al., 1999). The authors concluded that the results indicated changes in the nurses’ attitudes towards treating patients after stroke. Burton and Gibbon (2005) conducted a pragmatic, randomized, controlled study that aimed to evaluate whether expanding a specialist nursing role to give outreach education and support to stroke patients and carers after discharge from hospital was effective in promoting recovery. The study is the only study identified that measured the effect of an educational intervention for nurses on patient outcomes (primary outcome; detect a reduction in the prevalence of depressed mood assessed by the Nottingham Health Profile). Burton and Gibbon (2005) concluded that the intervention had substantial benefits for patients. Booth et al. (2005) conducted a quasi‐experimental study to measure the effect of a seven‐hour formal educational programme (lectures, simulated patient demonstration, video and experiential learning) that focused on therapeutic handling. They measured the effect of the educational programme using non‐participant observation and concluded that a change in therapeutic style had occurred (Booth et al., 2005).

The above studies differed in methods, duration, content, interventions and outcomes, which makes it difficult to draw conclusions. It therefore remains unclear what makes a clinically relevant educational intervention that strengthens the role and functions of inpatient nursing staff in stroke rehabilitation.

### 2.1 Aim

The aim was to assess the feasibility of a nursing educational intervention for inpatient stroke rehabilitation and its acceptability from the nursing staff’s perspective.

### 2.2 Design

Guided by the framework of the Medical Research Council (MRC) of the United Kingdom for developing complex interventions (Craig et al., 2008) and the Behaviour Change Wheel (BCW; Michie, Atkins, & West, 2014), we developed an educational intervention to strengthen the role and functions of nursing staff in inpatient stroke rehabilitation (Loft, Martinsen Woythal, et al., 2017; Loft, Martinsen, et al., 2017). An evidence‐ and theory‐based educational intervention (Loft, Martinsen Woythal, et al., 2017; Loft, Martinsen, et al., 2017) was systematically developed using these approaches that address the nursing staff’s capability (C), opportunity (O) and motivation (M) (COM‐B; Michie et al., 2014).

Conducting feasibility studies before the effect of an intervention can be evaluated is recommended (Richards & Hallberg, 2015) to ensure a clinically relevant intervention with a focus on practical issues and the intervention’s acceptability to the participants. Feasibility is addressed by considering contents, timing and dose. An intervention can be considered acceptable to staff if it addresses the problem appropriately, is easily adopted and followed and deals effectively with the challenge concerned (Richards & Hallberg, 2015).

Using mixed methods in a feasibility study can be useful and is recommended in the MRC framework (Creswell, 2014; Richards & Hallberg, 2015). In the present study, a convergence design (Creswell, 2014) was used to merge quantitative data derived from a questionnaire and qualitative findings from semi‐structured interviews and an intervention log document, which was maintained throughout the intervention period. The rationale for choosing a mixed‐methods design was the recognition of a need for different methods that can be combined to give a better understanding of the complex contextual environment of health care (Craig et al., 2008; Creswell, 2014; Richards & Hallberg, 2015).

#### 2.2.1 Intervention

The intervention tested in this feasibility study builds on a literature review, observations in a stroke unit and interviews with nursing staff and patients (Loft, et al., 2017; Loft, Martinsen Woythal, et al., 2017; Loft, Martinsen, et al., 2017).

Furthermore, observations were made at two other rehabilitation units to consolidate the results and to be able to describe usual care and a control group in a future trial. We used a theoretical and evidence‐based framework and selected two target behaviours: get nursing staff to work systematically with a rehabilitative approach and get nursing staff to work deliberately and systematically with the patient’s goals. The educational programme called *Rehabilitation 24/7* was delivered over 7 weeks (March–May 2016) in a stroke rehabilitation unit, which comprised 15 beds.

All Registered Nurses (RNs) and nurse assistants (NAs) working in the stroke rehabilitation unit participated in the 7‐week education programme, except for the substitute nursing staff. In broad terms, the intervention consisted of group education and training, training in practice and materials given as a feedback and reflection tool and as educational material (Table 1).

**Table 1:** The TIDieR (Template for Intervention Description and Replication) checklist (Hoffmann et al., 2014)

Item number		
1.	Brief Name Give the name or a phrase that describes the intervention	*Rehabilitation* 24/7
2.	Why Describe any rationale, theory, or goal of the elements essential to the intervention	The *Rehabilitation* 24/7 educational programme was developed using the Behaviour Change Wheel approach by addressing the capability (C), motivation (M) and opportunity (O) of the nursing staff in order to achieve the desired behaviour change. Overall, the programme aimed to optimise the rehabilitation of patients with stroke by strengthening the role and functions of nursing staff in inpatient stroke rehabilitation. Using a theoretical and evidence‐based framework, two target behaviours were selected: get nursing staff to work systematically with a rehabilitative approach, and get nursing staff to work deliberately and systematically with the patient's goals
3.	What Materials: Describe any physical or informational materials used in the intervention, including those given to participants or used in intervention delivery or in the training of intervention providers. Give information on where the materials can be accessed (e.g., online appendix, URL)	Rehabilitation 24/7 included: (1) *Rehabilitation* 24/7 script for the three workshops (for the educators) (2) Log book includes the following: Space for the participants to describe their reflections, observations, goals, etc., stickers illustrating main points from the theoretical presentations, explanations of the different tasks, etc. (for the participants) (3) Awareness‐raising posters illustrating individuals' goals, group discussions related to the theoretical presentation from all the workshops ‐ "what will we prioritise for further development in the unit" (for the participants) (4) A video illustrating a good rehabilitation situation (produced by and for the working group) (5) A video illustrating a situation of a nurse working with her individual set goals (produced by and for the working group)
4.	Procedures: Describe each of the procedures, activities and/or processes used in the intervention, including any enabling or support activities	*Rehabilitation* 24/7 consisted of the delivery of 14 behaviour change techniques to stroke inpatient rehabilitation nursing staff given by the *Rehabilitation* 24/7 educators. The educational programme consisted of three face‐to‐face workshops of three hours' duration with 2 weeks interval in between. The first work‐shop consisted of: Theoretical presentation about background to the study, rehabilitation (history, definition, evidence), stroke rehabilitation, patient involvement, patients' narratives Participants' reflections and discussion Introduction to and rehearsal of patient‐centred practice observation (PCPO) Presentation: Feedback The first tasks in practice: Patient‐centred observation 2–3 hr Reflections about the observation with reflection partner The second workshop consisted of: Participants reflect on their patient‐centred practice observation Theoretical presentation of nursing role and functions in inpatient stroke rehabilitation Discussions of and reflections about own practice and observations in groups and plenum, formulating goals for changes in practice, video showing examples of working with a rehabilitative approach The second tasks in practice: The nursing staff work with own individual goals and changes in practice. The third workshop consisted of: Theoretical presentation of goal setting in rehabilitation Role play in two distinct groups represented by the two professions. A framework was given to the groups to help them focus on how to verbalise the nursing staff's professional language. Analysis and evaluation of individual goals Poster review, discussion and plans for the future?
5.	Who given Describe the modes of delivery (e.g., face‐to‐face or by some other mechanism, such as internet or telephone) of the intervention and whether it was given individually or in a group	Criteria for *Rehabilitation* 24/7 educators: 1. A nurse with at least a master's degree 2. Another professional with at least a master's degree who had experience of facilitating processes of change in the healthcare system and competency development
6.	How Describe the modes of delivery (e.g., face‐to‐face or by some other mechanism, such as internet or telephone) of the intervention and whether it was given individually or in a group	Rehabilitation 24/7 was delivered in three face‐to‐face group session by the educators using a pre‐developed script
7.	Where Describe the type(s) of location(s) where the intervention occurred, including any necessary infrastructure or relevant features	*Rehabilitation* 24/7 was delivered at university hospital classrooms and training took place in the stroke unit. The programme aimed at the patients was delivered at the stroke rehabilitation unit
8.	When and How Much Describe the number of times the intervention was delivered and over what period of time including the number of sessions, their schedule, and their duration, intensity or dose	*Rehabilitation* 24/7 was delivered over 7 weeks and consisted of three workshops of 3 hr each. Between each workshop, tasks and training were performed in the stroke unit by the individual RN or NA or together with their reflection partner. There were 2 weeks between each workshop
9.	Tailoring If the intervention was planned to be personalised, titrated or adapted, then describe what, why, when and how	‐N/A
10.	Modifications If the intervention was modified during the course of the study, describe the changes (what, why, when and how)	‐N/A
11.	How Well Planned: If intervention adherence or fidelity was assessed, describe how and by whom, and if any strategies were used to maintain or improve fidelity, describe them	Fidelity of the intervention delivery was described using a log book by the Rehabilitation 24/7 educators
	Actual: If intervention adherence or fidelity was assessed, describe the extent to which the intervention was delivered as planned	Overall, the intervention was delivered as planned; however, not all participated in the reflection‐partner meeting

**Figure 1:**
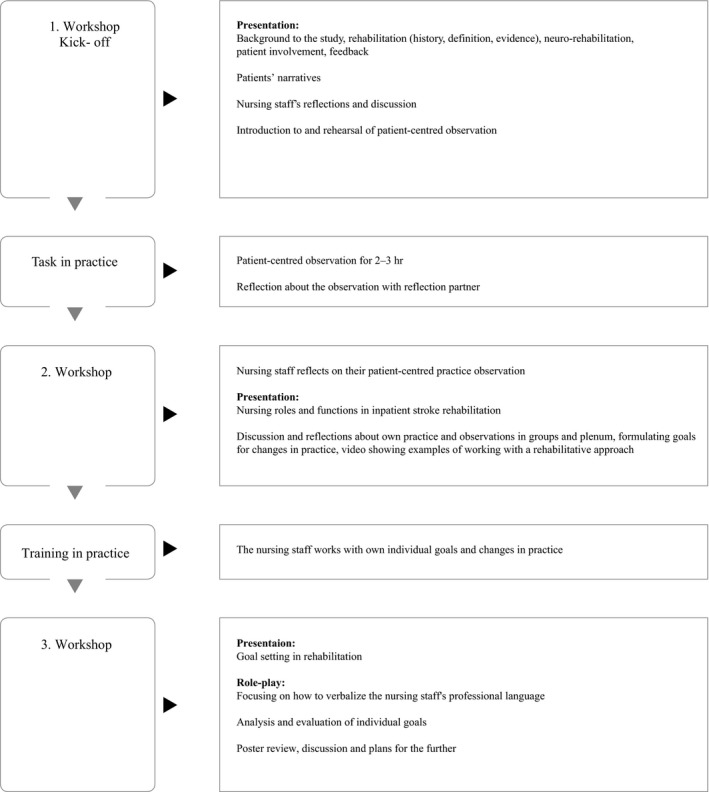
Flow chart showing the rehabilitation 24/7 educational programme

The nursing staff were purposively split into three groups with approximately 12 nurses in each. Considerations were given to age, educational level and degree of experience with stroke rehabilitation. Three group sessions of 3 hr each were conducted for each group. Two weeks were allowed in between the workshops and nursing staff carried out tasks and training as per their daily clinical practice. A flow chart of the programme is illustrated in Figure 1.

### 2.3 Sample/participants

The nursing staff in the rehabilitation unit consisted of RNs (*N* = 19) and NAs (*N* = 18). In total, 36 participated in the educational programme. One NA did not participate as she would retire shortly. All participants in the educational programme were asked to answer the questionnaire immediately after programme completion. The questionnaire was completed by 31 participants.

For the interviews, RNs (*N* = 6) and NAs (*N* = 4) were selected to obtain a purposive sample to ensure a broad and varied perspective (Kvale & Brinkmann, 2014). Thus, RNs and NAs with different seniority, experience and age were selected. The participants were contacted by the first author in collaboration with the managers of the stroke unit. The principle of data saturation guided participant sampling. After 10 interviews, the two main authors agreed that saturation had been reached.

### 2.4 Data collection

The data were collected between February ‐ July 2016.

#### 2.4.1 Questionnaire

A 6‐item questionnaire was developed to examine feasibility and acceptability. Answers were given on a 4‐point Likert scale which ranged from totally agree, agree, disagree and totally disagree.

#### 2.4.2 Interviews

The interviews were conducted using a semi‐structured interview guide (Kvale & Brinkmann, 2014). The guide covered participants’ perceptions of the programme in relation to its feasibility and acceptability. All of the interviews were conducted in an outlying office in the ward and lasted between 37 and 50 min (a mean of 44.6 min). The interviewer, who was an experienced senior nursing researcher, was unknown to the participants and had not been involved in the development or delivery of the programme. The interviews were digitally recorded and transcribed verbatim.

### 2.5 Ethical considerations

According to the Danish National Committee on Health Research Ethics, only studies defined as biomedical research studies require approval. Therefore, this study was not registered under the Committee Act (Protocol Number.: H‐2‐2014‐038). This study was registered with the Danish Data Protection Agency. The ethical principles of the Declaration of Helsinki were followed. The participants given informed consent to participate in the study.

### 2.6 Data analysis

#### 2.6.1 Questionnaire

The questionnaires were analysed using descriptive statistics summarized as percentages and numbers using IBM SPSS Statistics (IBM SPSS Version 22.0).

#### 2.6.2 Interviews

The qualitative interview data were analysed using deductive content analysis according to the method described by Elo and Kyngäs (2008). A structured categorization matrix (Elo & Kyngäs, 2008) was developed based on the intervention categories, that is content, functions, planning and relevance. The matrix was used as a lens with which to analyse the interviews, and they were themed to facilitate answering the research questions about the feasibility of the educational programme, its acceptability to the nursing staff and intervention‐related functions, for example theory, tasks and material.

Three members of the research team independently read the transcript multiple times to become familiar with the content and to acquire an overview of the texts. Then, the transcripts were reviewed for content. Text that corresponded to the matrix categories was coded and transferred to the matrix. A description for each category, representative of a manifest level, was then made. NVivo^®^ software (QSR International Pty Ltd., Victoria, Australia) was used to obtain an overview and to facilitate a systematic approach to analysing the interviews.

#### 2.6.3 Mixed‐method analysis

The results from the questionnaire were compared with the findings from the interviews in a side‐by‐side comparison to investigate whether they were convergent and/or expanded (Creswell, 2014).

### 2.7 Validity and reliability

The trustworthiness of qualitative studies can be assessed from their credibility, transferability, conformability and dependability (Elo et al., 2014). We have presented the audit trail of the study and described how we established credibility by recruiting participants in the study who had the ability and willingness to share their experiences and perceptions on the subject under study and who had different backgrounds and experiences. The sample size was determined by data saturation (Elo et al. 2014) established after the preliminary analysis of the first eight interviews, and the findings were further confirmed after having recruited two more participants. Thus, credibility was established. Through a detailed description of the data analysis and structure of the categories with confirmation from three authors, the dependability of the research findings was established. To facilitate transferability, a clear description of the context, selection and characteristics of the respondents, data collection and process of analysis was presented. Reflexivity was addressed by the researchers in an ongoing process with utmost attention to the effects of our pre‐understanding. The research team consisted of researchers from different institutions, clinical practices and professions.

In this study, we developed a questionnaire where the questions on the feasibility and acceptability of the intervention were simple and easy to answer. However, the questionnaire was tested only on a small sample size (*N* = 5) before being used in the present study. Based on this test, no changes were made. We reported details of our sampling method for the qualitative data. The sampling method was purposive, and sampling was stopped when the first and the last author agreed that data saturation had been reached. Furthermore, trustworthiness in the content analysis was sought through a concise description of the analytical process and we sought validation of the findings from the interviews through the research team’s continuing discussions until consensus was achieved.

## 3 RESULTS/FINDINGS

For characteristics of the sample for the questionnaire and the interviews (Table 2). The results of the quantitative and qualitative analysis will be presented separately. This presentation will be followed by a comparison in a mixed‐method section.

**Table 2:** Sample characteristics for the questionnaire and interviews

Variable	*N* (%) Questionnaire	*N* Interviews
Professional group		
Registered Nurse	16 (51.5)	6
Nurse assistant	15 (48.5)	4
Sex		
Male	2 (6.5)	1
Female	29 (93.5)	9
Years since education		
<2 years	7 (22.6)	2
2–5 years	1 (3.2)	0
>5 years	23 (74.2)	8
Supplementary education		
Yes	11 (35.5)	3
No	20 (64.5)	7
Current employment		
<2 years	13 (42.0)	3
2–5 years	6 (19.3)	1
>5 years	12 (38.7)	6
Experience working with stroke rehabilitation		
<2 years	8 (25.8)	3
2–5 years	4 (12.9)	0
>5 years	19 (61.3)	7

### 3.1 The quantitative results

The questionnaire response rate was 94% and 100% of the sample either agreed or totally agreed that the educational programme was interesting (question#1) and relevant (question#2) for their clinical practice. Most of the nursing staff found that the lectures in the workshops were educational (question#3) as 96.8% either agreed or totally agreed. The professional level of the workshops (question#4) was considered appropriate for the main part as 93.6% agreed or totally agreed on this. In total, 96.8% of the participants agreed or totally agreed that the educational programme was well planned (question#5). Similarly, 96.8% either agreed or totally agreed that variation between active participation, exercises and presentations was well planned (question#6; Table 3).

**Table 3:** Acceptability of the educational programme % (*N*)

	Totally Agree	Agree	Disagree	Totally disagree
1. Lectures were interesting	22.6 (7)	77.4 (24)	0.0	0.0
2. Relevance to my daily practice	32.3 (10)	67.7 (21)	0.0	0.0
3. Lectures were educational	32.3 (10)	64.5 (20)	3.2 (1)	0.0
4. The professional level was appropriate	19.4 (6)	74.2 (23)	6.4 (2)	0.0
5. The workshops were well planned	25.8 (8)	71.0 (22)	3.2 (1)	0.0
6. Variation between active participation, exercise and presentations was well planned	16.2 (5)	80.6 (25)	3.2 (1)	0.0

### 3.2 Qualitative findings

The qualitative findings are revealed in three overall categories that relate to feasibility, acceptability and intervention functions. The intervention functions refer to the interviewees’ detailed descriptions of the elements of the programme.

#### 3.2.1 Feasibility

Feasibility was assessed by addressing whether the programme could be given as planned and whether its duration and extent were appropriate. Thirty‐one out of 36 participants were present for the entire educational programme. Sickness, vacations and personal reasons were given as explanations for absence. Participation was mandatory. However, two members of the nursing staff did not meet and they gave no reasons for their absence.

The education was experienced as meaningful and as a basis for change in clinical practice. However, the nursing staff found that having to continue with clinical work while attending the workshop sessions put them under pressure, especially as they had to stand in for absent nursing staff:

*While it … has been … challenging …, some people stop, some went on maternity leave. It has been enormous pressure to have … it … on top of everything, yet we have done it and hung in there. I think it has been very good. Everyone thinks the same. Those who had been on the course were allowed to go on the course and those who remained had to give an extra hand. The following week*,* the roles were reversed. I think it is very good that it was the whole group and not just some who went*. (RN1)

The support and participation of management were seen as essential to facilitating involvement and for the opportunity of conducting the education programme. Some found it challenging to adapt from a busy morning caring for patients to the relative peace and quiet of sitting through a workshop. Most were satisfied with the structure, although some said that they would prefer if an entire day were devoted to the workshops. The participants were satisfied with the length of the programme as it allowed for in‐depth exploration of all components of the stroke rehabilitation process.

The patient‐centred observation (PCPO) exercise was completed as planned though the nursing staff found it challenging to be both observers and non‐participants in a daily clinical practice setting. Nevertheless, all managed to complete the exercises successfully even though it was difficult to set aside time to reflect on the exercises. As a result, some did not complete the reflection‐partner meeting before the next workshop.

### 3.3 Acceptability

The programme was welcomed and described as meaningful. Before it started, some participants said that they found it difficult to imagine what to expect and that there was some scepticism:

And those who were not so hot on the idea initially came around to it. So I think after we first got started, something positive came of it. (NA 2)

The programme was described as “meaningful” from both an individual and a group perspective. It was also described as having helped strengthen the “shared language” of the nursing group internally and in relation to patients, families and interdisciplinary collaborators:

It means that there is a shared language. It is very consistent now what happens here. We all focus on rehabilitation; it is really good. (NA 3)

The programme had given advanced professional knowledge such as greater knowledge about the concept of stroke rehabilitation, what is involved in having a stroke and what inpatient rehabilitation means to patients and their families. This helped nursing staff to prioritize clinical practices in an attempt to meet patients’ needs.

From the participants’ perspectives, their role and functions were clearer because of increased understanding:

*What I take from this is that I have reflected more on how important we are at a very basic level for the patients*—*what we do is much more important than I thought. Maybe I already knew it. I have just not been able to put it into words.* (RN4)

Thus, the contribution required from the nursing staff was more efficiently articulated.

### 3.4 Intervention functions

The participants greatly appreciated the variation between information, participation during discussions and exercises. The theoretical lectures were described as “meaningful” and “relevant”; the lectures included theory that was known, but had been forgotten, and newer concepts. The theoretical input made it possible for the participants to frame their daily experiences and express these in words.

The nursing staff was enthusiastic about the exercise where they had to carry out a PCPO. This was described as an exercise that involved acquisition of knowledge and personal reflection about putting oneself in the patient’s place, with focus on communication between the nursing staff and patients and among the nursing staff themselves. It also emphasized the actions, roles and functions of the nursing staff:

We got this unique chance to observe each other and put ourselves in the patient's place. It was, indeed, an eye‐opening experience for most of us. (RN5)

The observation exercise was a unique sensory experience not previously experienced, and it involved heightened awareness of various sounds, smells and emotions, which were a source of further reflection. Through the observation exercise, the participants became attentive to their own actions and how these affected the patient.

Especially at the beginning, some were nervous about being observed, but they soon became aware that the objective was not to target the actions of any one individual. The subsequent analysis and reflection‐partner meeting were always conducted in an acknowledging way with newly acquired feedback providing a framework for the ensuing conversation.

Patients’ narratives were described as making an emotional impression on the nursing staff, providing knowledge about how they experienced being admitted and how they experienced the nursing staff The nursing staff had not previously been aware of these perspectives:

It was also very exciting because the educator read patients’ stories, what they had said and how they perceive us as staff. It was hard. (RN6)

The intervention‐related content concerning feedback was described as meaningful on several levels. The participants had developed a new perspective on how feedback benefitted the patient–nurse relationship. Feedback also facilitated communication among nursing staff and interdisciplinary collaborators and thus had a positive impact on the nursing staff’s approach to everyday practice:

*Yes, it was really good, the feedback part. Everyone just talked about it and you could feel in the unit that they began to consider how to talk to each other*.*. the feedback could be among colleagues, but it could also be patient‐colleague or patient‐nurse, so it can involve crisscrossing.* (RN7)

For some, role‐play transgressed personal boundaries at the start, but it was described as educational and a fun way to train and operationalize newly learned theories.

The nursing staff’s individual goals were written on posters as were also summary points from the workshops, and the posters were displayed in their office. This was described as creating a sense of community among the nursing staff. At the same time, it given a record of what had happened and what still needed to be done. The logbook that was given to the participants on commencement of the programme was described in positive terms and became an important tool throughout.

#### 3.4.1 Mixed‐method analysis

Overall, there was a high level of agreement between the results of the quantitative and the qualitative analyses (Table 4). The high level of agreement that the lectures were interesting was convergent, but PCPO also given a more detailed description of the aspects that had had an impact. This demonstrated that PCPO had an impact, but the role‐play activity, patient narratives and the theoretical lectures were also mentioned as beneficial. Some elements, such as the video, were not mentioned as having made a particular impression.

**Table 4:** Joined display outcome of the merged quantitative and qualitative results

Quantitative results	Quantitative results	Outcome of the merged findings
Lectures were interesting	The educational programme consisted of several components that were described as interesting. Especially, the patient‐centred observation was described as a unique sensory experience that emphasized the actions, role and functions of the nursing staff and the communication between patients and the nursing staff. The role play and theoretical lectures were described as being interesting.	Convergent and expanded
Relevance to my daily practice	The lectures were relevant to the nursing staff's clinical practice.	Convergent and expanded
Lectures were educational	Professional levels were described as increased, providing knowledge about stroke rehabilitation and the nursing staff's professional role and functions. Lectures strengthened the nursing staff's shared professional language.	Convergent and expanded
The professional level was appropriate	The lectures consisted of theory that was known, but had been forgotten, and newer concepts. The theory made it possible for the nursing staff to frame their daily experience and express it in words.	Convergent
The workshops were well planned	The length of the educational programme was suitable and worthwhile, even though it was a challenge to make it all work. The size of the groups was appropriate. Patient‐centred observation was a challenging task to complete, but it was successful. Not all of the nursing staff participated in the reflection‐partner meeting. More time for the workshops was demanded so that they could be more rewarding.	Convergent and expanded
Variation between active participation, exercises and presentations was well planned	Both information about and participation in the discussions and exercises were greatly appreciated. The theoretical presentation was challenging, but acceptable, as it was given in short doses and mixed with active participation.	Convergent and expanded

The lectures received scores on their relevance to the nursing staff’s clinical practice. While this finding was convergent, it also expanded the interviewed nursing staff’s sparse descriptions of the relevance of practice.

Nursing staff noted that the lectures were educational. This was convergent and expanded on the staff’s descriptions of how professional levels were enhanced and how the lectures strengthened the nursing staff’s sense of having a shared language and a deeper understanding of their role and functions.

It was not explicitly expressed in the interviews whether the professional level was appropriate; however, it seemed so from the nursing staff’s descriptions because the theory was described as making it possible to frame daily practice; moreover, the theory consisted of both new knowledge and knowledge that was previously known but had been forgotten. This was in agreement with the high score on the appropriateness of the level of the programme. Hence, 97% considered the educational programme to be well planned. This was convergent with and expanded on by descriptions from the qualitative analysis. The group size and duration of the programme were described positively, whereas descriptions obtained from the interviews elaborated on how it had, after all, required additional resources from the staff to make everything work in the daily practice. Even though the nursing staff expressed satisfaction with the workshops, they also suggested that they should be longer. Descriptions from the interviews showed that it was difficult for the nursing staff to operationalize the reflection‐partner meetings, which is why the meetings were not conducted for all staff members.

Variation between active participation exercises and presentations was found to be well planned and had a high positive agreement score. This was in agreement with findings from the interviews. For instance, the inclusion of different activities made it easier for them to concentrate on the theoretical lectures.

## 4 DISCUSSION

The results show that the intervention was feasible despite its complexity. Findings from the interviews show that the intervention required extra resources from the staff as they had to work faster than usual to make it all possible. Despite this, the nursing staff described the intervention as being worth the effort, meaning they found it fulfilling and relevant. Furthermore, the results show a high level of acceptance of the educational programme. According to Fan, Sidani, Cooper‐Brathwaite, and Metcalfe (2013) and Sidani (2008), this could contribute to improved attendance and compliance among participants and strengthen the feasibility. We believe that the high level of feasibility of our programme was obtained because of its high level of acceptability to the nursing staff.

The programme was developed using a systematic approach, that is the BCW (Michie et al., 2014), where all the elements, techniques and forms of delivery were based on analyses. In this approach, implementation was considered part of the development from the very beginning. According to our results, the programme elements seemed to be working well together. This is in agreement with other studies where workshops were also reported as being feasible in most settings (Grimshaw et al., 2001). Regarding implementation science, there is agreement that multifaceted strategies are more likely to have a positive outcome (Grimshaw et al., 2001). Thus, the multifaceted, tailored strategy we used may be one of the main reasons for the positive results.

Using the MRC framework and the BCW guided us to develop an intervention that takes into consideration barriers and facilitators for the working staff’s changing behaviour in relation to working systematically with patients’ goals and with a rehabilitative approach. The initiative was based on evidence and theory yet had a practical foundation. We took into consideration the two different professional groups, RNs and NAs, and that we had to find appropriate levels for both. Furthermore, we considered the didactics appropriate for educating adults. According to Entwistle (2009), it is well known from early behaviourist theories that skills are developed through practice and that it is important for improving skills and knowledge that practices are varied. Also, Entwistle (2009) described how attention and memory are related and that long‐term memory (LTM) is linked to relevant areas of knowledge and expertise. Episodes and past events are often associated with sounds, smells and feelings stored in what is called episodic LTM, whereas knowledge and ideas are stored in semantic LTM. These two long‐term memories are closely linked; it is possible to trig the semantic LTM through the episodic LTM. These considerations are integrated into our didactic considerations about teaching nursing staff through both theory and practice and addressing both semantic and episodic memory.

The participants found the educational level appropriate. They described how they gained new knowledge and how this could frame their practice. They also noted that they now had a shared language, which underscored the relevance of the programme content. A few other studies (Burton & Gibbon, 2005; Forster et al., 1999; Jones et al., 1998) have also focused on strengthening the role and functions of nursing staff in the inpatient rehabilitation of stroke patients through educational intervention. However, in these previous studies, acceptability and feasibility were not described, which makes it difficult to draw comparisons between our study and others’.

In previous studies on educational interventions for nursing staff in other clinical areas, barriers to practice change were identified. These barriers included conditions of service, relationships with colleagues and support from management (Dick, Lewin, Rose, Zwarenstein, & Van Walt, 2004), prioritizing patient care, avoiding burdening colleagues and lack of management and collegial support (Berthelsen & Hølge‐Hazelton, 2016). In our study, these barriers were also mentioned as challenging. However, we tried to address these barriers and the fact that the entire nursing staff participated in the programme was given as a reason for overcoming these barriers.

The interviews showed that the participants noticed management’s support and had a positive attitude towards the active participation of the two wards nurses, which was also considered necessary if their new knowledge should be operationalized. From the very beginning, the care management team was involved at several levels in developing the intervention and planning the implementation strategy. The high, positive score for both feasibility and acceptability may (also) be related to management’s involvement as a complex educational intervention demands support from the management of an organization since the intervention requires hours of work from the nursing staff. Management’s support of the programme may have had a positive effect on attendance and retention. In a study by Luker et al. that evaluated a process of implementation of a mobilization intervention in a stoke unit, the authors found that the leaders’ attitudes and support were important for delivering the intervention and ensuring the staff’s engagement.

The participants indicated that variation between active participation, exercises and presentations was well planned. The PCPO exercise made a significant emotional and educational impression. The nursing staff was usually busy, and it was therefore a challenge to observe without having to act. Particularly the fact that the staff should not focus on finding solutions could be a reason for the value ascribed to the exercise and the great impression it made.

The reflection‐partner meeting was mentioned in both the interviews and the logbook as not having been undertaken by all the participants. This may have been so because this element differed from the other elements in that the participants had to take responsibility themselves for meeting. This issue raises the general point about the problems of getting participants to take responsibility for their learning and getting participants to understand that a project may be important for their daily work, their professional role and functions and, in particular, their patients. The fact that not all of the participants completed their reflection‐partner meeting may be explained by the difficulty of arranging this meeting in an already busy practice, especially when the participants, not a leader or other stakeholders, were expected to make it a priority. As the results showed that 100% of the participants found the education programme interesting and relevant, we interpret that the difficulty in participating in a reflection‐partner meeting was not due to lack of interest from the nursing staff but to the fact that we had not identified all possible barriers. Investigating changing professional practice through an educational intervention, Dick et al. addressed the issue that participants working in a system with many top‐down approaches would be unfamiliar with the expectation of self‐reflection and problem‐solving. Although the setting was different, this explanation is worth considering when staff is working in a healthcare system under pressure, where they are constantly introduced to new guidelines and are required to implement these guidelines in the context of a hierarchy.

### 4.1 Study limitations

We chose to have an unequal sample size as the questionnaire was completed by a larger number of nurses than participated in the interviews. This could mean that the results were difficult to merge (Creswell, 2014). However, the merged data were consistent.

One limitation of this study is its generalizability. The study was developed and tested in one context, which may raise questions about its feasibility and acceptability in other contexts. However, as part of the development phase, the first author was a participant observer in two other stroke units to ensure that the identified facilitators and barriers were not just local phenomena.

## 5 CONCLUSION

*Rehabilitation 24/7* was a feasible and acceptable educational programme in a clinical inpatient stroke rehabilitation setting. Using a mixed‐method design given in‐depth understanding of the educational programme’s feasibility and its acceptability to nursing staff. This intervention was developed in the field of stroke rehabilitation; the way of developing the intervention by identifying needs, barriers and facilitators is recommended for other healthcare areas where there is a need and desire to increase knowledge and change professional behaviours. Based on the positive feasibility and acceptability evaluation, the conclusion is that only minor changes are needed. For instance, we will make sure that the reflection‐partner meeting is scheduled. Furthermore, we will devote a little more time to the theoretical presentations by taking out the video element that was not mentioned as beneficial by any of the participants. The intervention was developed and delivered in an already existing context. Additional staff hours were required to conduct the educational intervention, but we illuminated that it is possible to make behavioural changes for a collective staff group in an already existing context. Consequently, in keeping with the MRC framework, it would be relevant to investigate further the causality between the intervention and the intended effect.

## CONFLICT OF INTEREST

No conflict of interest has been declared by the author(s).
